# Association of Axial Length and Refraction with Near Horizontal Heterophoria in Chinese Children: An Observational Cross-Sectional Study

**DOI:** 10.1155/2022/7549851

**Published:** 2022-05-31

**Authors:** Xiaoqin Chen, Yanglin Jiang, Qian Fan, Lihua Li, Wenli Lu, Yan Wang

**Affiliations:** ^1^Tianjin Eye Hospital and Nankai University Eye Institute, Tianjin Key Lab of Ophthalmology and Visual Science, Tianjin Eye Institute, Tianjin, China; ^2^Nankai University Affiliated Eye Hospital, Nankai University, Tianjin, China; ^3^Clinical College of Ophthalmology, Tianjin Medical University, Tianjin, China; ^4^Tianjin Eye Hospital Optometric Center, Tianjin, China; ^5^Department of Epidemiology and Health Statistics, Tianjin Medical University, Tianjin, China

## Abstract

**Purpose:**

To evaluate the association of near heterophoria with refraction and axial length (AL) in Chinese school children.

**Methods:**

This school-based cross-sectional study included 15,081 Chinese primary school children (grades 1–6) examined during 2017. Near heterophoria was measured at 33 cm using the Maddox rod and prism test. Noncycloplegic refraction and AL were also measured. A generalized additive model with a Gaussian link was used to determine the association of near heterophoria with refraction and AL. Analyses were adjusted for age to account for differences in the age distribution of the sample.

**Results:**

Overall, data were analyzed for 11,013 students ranging in age from 6 to 13 years. The most common type of near heterophoria was exophoria (64.96%), the proportion and value of which increased according to grade. Exophoria accounted for 62.53% (2,328/3,723), 65.03% (2,501/3,846), and 67.51% (2,325/3,444) of near heterophoria cases for grades 1-2, grades 3-4, and grades 5-6, respectively. Prism diopter (PD) values for near heterophoria in these grades were −6.30 ± 3.69, −6.81 ± 4.01, and −8.32 ± 5.12 PD, respectively. The average spherical equivalent (SE) in children with orthophoria was 0.23 D and 0.25 D lower than those in children with exophoria and esophoria, respectively (*P* < 0.001). The mean AL in children with orthophoria was 0.11 mm shorter than that in children with either exophoria or esophoria (*P* < 0.001). Near heterophoria exhibited a significant correlation with refraction and AL, irrespective of age.

**Conclusions:**

Exophoria represents the most common type of near heterophoria in children. Children with more severe near heterophoria, whether exophoria or esophoria, exhibited a higher degree of myopia and longer AL than those with relatively less severe near heterophoria. These results highlight the need for further, long-term investigation regarding the role of near heterophoria in visual development.

## 1. Introduction

Myopia is among the leading causes of visual impairment worldwide, representing a global public health concern due to its increasing prevalence. By 2050, an estimated 2.5 billion and 1 billion people will be affected by myopia and high myopia, respectively [1]. Previous studies have reported that the prevalence of myopia (>80%) is higher among children in Southeast Asian countries than in other regions, especially China [2, 3]. Myopia has also been associated with several ocular complications, including retinal damage, cataracts, and glaucoma [4–7]. Research indicates that the etiology of myopia is multifactorial and may be related to near work and heterophoria status [8–14].

Heterophoria is a latent deviation of the eyes that is only revealed in the absence of an adequate stimulus to fusion [15]. Although several studies have reported an association between myopia and near heterophoria, the significance of this association remains unclear [16–20]. One study conducted in the US reported that children with exophoric heterophoria >3 prism diopters (PD) or esophoric heterophoria >1 PD exhibited an increased risk of myopia, further noting that children with near esophoria experienced faster myopic progression than children with orthophoria or exophoria [13]. While several studies have also reported an association between near heterophoria and the amount of ametropia [14, 19–21], others have found no association between myopia and near heterophoria [22, 23], and few large-scale population-based studies have focused on near heterophoria.

Given the high prevalence of myopia in the Chinese population [24–26], some authors have argued that near heterophoria may influence the risk of myopia. Therefore, in the present study, we aimed to investigate the distribution of near heterophoria as well as its association with refraction in Chinese school children. Moreover, we utilized a comparative design as an alternative to the traditional population-based design to verify the relationship between refraction and axial length (AL) in a large sample of participants with different degrees of heterophoria.

## 2. Materials and Methods

### 2.1. Participants

This cross-sectional study included 15,081 school children (aged 6 to 13 years) who underwent assessments in Tianjin, China, in 2017. Written informed consent was obtained from at least one parent, and each child provided verbal consent prior to visual screening and participation in the study. The study was conducted in accordance with the Declaration of Helsinki and was approved by the Ethics Committee of Tianjin Eye Hospital (2021020). This trial was registered on ClinicalTrials.gov (NCT04073238) and followed the Strengthening the Reporting of Observational Studies in Epidemiology (STROBE) guidelines.

### 2.2. Visual Examination

All students underwent a detailed visual examination, including slit-lamp examination, fundus examination, and measurements of intraocular pressure using noncontact tonometry (CT-80, Topcon Yamagata Co, Ltd, Tokyo, Japan). The corneal reflections test (Hirschberg test), unilateral cover test, and alternate cover test were also performed. Children with abnormal findings or strabismus were excluded from the study. The degree of near heterophoria was measured using the Maddox rod and prism test [27]. Briefly, a red Maddox rod was placed in front of the right eye, following which the children were asked to report the relative position of the Maddox rod streak with respect to a torchlight presented at a distance of 33 cm. The distance between the Maddox rod streak and the torchlight was neutralized using prisms, and the values were recorded. Heterophoria measurements were repeated three times for each child, and the average values were used for the analysis. Heterophoria was measured daily, and children who wore glasses underwent assessments while wearing their normal glasses. Noncycloplegic autorefraction and AL were measured using an autorefractor (KR-8900, Topcon Yamagata Co, Ltd, Tokyo, Japan) and an optical biometer (AL-Scan, Nidek Co., Ltd., Aichi, Japan), respectively. Both tests (refraction and AL) were repeated five times, and the average values were used for the analysis. All measurements were obtained by experienced optometrists.

Orthophoria was defined as the degree of heterophoria between −2 PD and +2 PD. Myopia was defined as a spherical equivalent (SE) ≤−0.50 diopters (D), while hyperopia was defined as an SE ≥+2.00 D.

### 2.3. Statistical Analyses

As we observed a strong correlation between right and left eye values for SE and AL, analyses were performed using data from right eyes only (*r* = 0.847, *P* < 0.001; *r* = 0.842, *P* < 0.001, respectively). All statistical analyses were performed using the MGCV package in R software (version 3.6.2). The normality of the data was assessed using the Shapiro–Wilk test. AL was compared among children with different heterophoria status using the one-way analysis of variance (ANOVA). Non-normally distributed refraction data for different types of near heterophoria and differences in near heterophoria among different school grades were compared using the Kruskal–Wallis H test. The Spearman rank correlation coefficients were used to evaluate the relationship between age and near heterophoria. Associations of near heterophoria with SE and AL were analyzed using generalized additive models (GAMs), and the smooth terms were represented as penalized thin-plate regression splines. In model 1, a GAM with a Gaussian link was applied to evaluate the associations of near heterophoria with SE and AL. The natural spline smoothing functions of near heterophoria were used as the independent variable, while SE or AL was used as the dependent variable. In model 2, the natural spline smoothing functions of near heterophoria and age were entered for covariate adjustment. The level of statistical significance was set at *P* < 0.05.

## 3. Results

### 3.1. Demographic Characteristics, Heterophoria Distribution, and Mean Values of Near Heterophoria in the Study Population

A total of 15,081 school children ranging in age from 6 to 13 years were examined, including 376 (2.49%) children with strabismus. Children with missing data, significant systemic illnesses, and strabismus were excluded, resulting in a total enrollment of 11,013 children (52.6% male) with a mean age of 9.26 ± 1.73 years. A total of 1,559 (14.16%) students wore glasses regularly.  Table 1 shows the basic demographic parameters and important ocular parameters. There were significant differences in age, gender, SE, and AL between included and excluded subjects (*P* < 0.05). However, no significant difference was found in heterophoria between the two groups (*P*=0.197).

Children with orthophoria constituted 23.30% (2,566/11,013) of the sample. Exophoria and esophoria were observed in 64.96% (7,154/11,013) and 11.74% (1,293/11,013) of children, respectively. The mean values of near heterophoria were −0.06 ± 0.50 PD, −7.15 ± 4.41 PD, and 5.74 ± 4.54 PD in children with orthophoria, exophoria, and esophoria, respectively. The Shapiro–Wilk test revealed that the overall distribution of near heterophoria was non-normal (*P* < 0.001).

**Figure 1:**
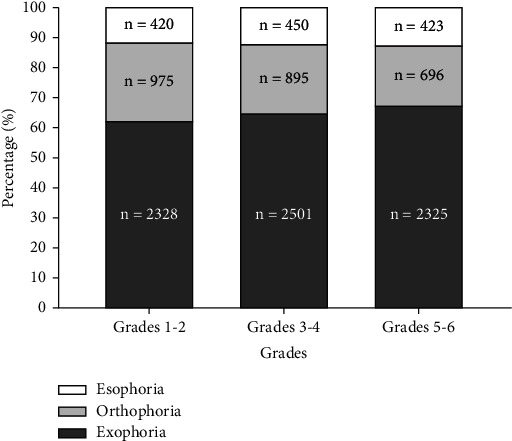
Distribution of different types of near heterophoria according to grade level.

Children were divided into three groups according to grades (grades 1-2, grades 3-4, and grades 5-6).  Figure 1 shows that exophoria was the most common form of heterophoria, accounting for 62.53% (2,328/3,723), 65.03% (2,501/3,846), and 67.51% (2,325/3,444) of cases in grades 1-2, grades 3-4, and grades 5-6, respectively. The proportion of exophoria increased, while that of orthophoria decreased, as grade level increased. The average values of near heterophoria were −3.38 ± 5.14 PD, −3.87 ± 5.52 PD, and −4.76 ± 7.24 PD in grades 1-2, grades 3-4, and grades 5-6, respectively.

**Figure 2:**
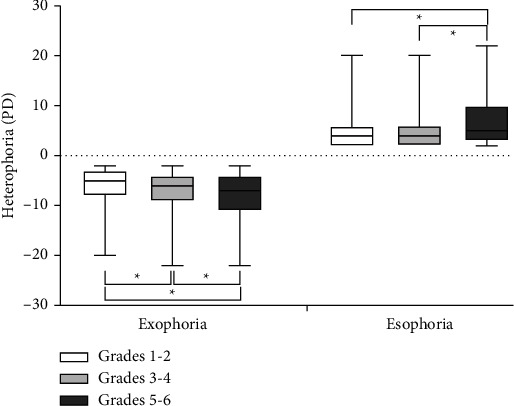
Degree of near heterophoria according to grade level. The degree of near heterophoria was compared with different grade-level groups using the Kruskal–Wallis *H* test (*P* < 0.001). ^*∗*^Significant differences (*P* < 0.001).

As shown in Figure 2, the average values of near heterophoria were −6.30 ± 3.69 PD, −6.81 ± 4.01 PD, and −8.32 ± 5.12 PD in children with exophoria in grades 1-2, grades 3-4, and grades 5-6, respectively. The degree of exophoria increased as grade level increased, and there were significant differences among the groups. The average degrees of near heterophoria were 5.04 ± 3.84 PD, 5.00 ± 3.64 PD, and 7.23 ± 5.57 PD in children with esophoria in grades 1-2, grades 3-4, and grades 5-6, respectively. There were also significant differences in the degree of esophoria between grades 1-2 and grades 5-6, and between grades 3-4 and grades 5-6.

### 3.2. Distribution of Near Heterophoria in Children with Different Degrees of Refraction

Myopia was observed in 31.24% (1,163/3,723), 54.21% (2,085/3,846), and 77.96% (2,685/3,444) of children in grades 1-2, grades 3-4, and grades 5-6, respectively. Emmetropia was observed in 67.79% (2,524/3,723), 44.88% (1,726/3,846), and 21.34% (735/3,444) of children in grades 1-2, grades 3-4, and grades 5-6, respectively. Rates of hyperopia were only 0.97% (36/3,723), 0.91% (35/3,846), and 0.70% (24/3,444) in grades 1-2, grades 3-4, and grades 5-6, respectively.  Table 2 shows the distribution of near heterophoria according to differences in refraction.

### 3.3. Refraction and AL in Different Types of Near Heterophoria

SE and AL differed significantly between different types of near heterophoria (*H* = 21.328, *P* < 0.001; *F* = 9.098, *P* < 0.005, respectively). The average SE in children with orthophoria was 0.23 D lower than in children with exophoria and 0.25 D lower than in children with esophoria (*P* < 0.001). Mean AL was 0.11 mm shorter in children with orthophoria than in children either with exophoria or esophoria (*P* < 0.001) (Table 3).

### 3.4. Associations of Near Heterophoria with SE and AL

**Figure 3:**
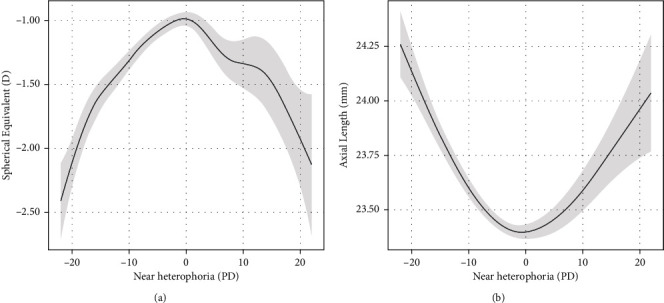
(a) Nonlinear relationship between near heterophoria and SE. The gray area represents the 95% confidence interval of the estimated SE. (b) Nonlinear relationship between near heterophoria and AL. The gray area represents the 95% confidence interval of the estimated AL.

Figure 3 depicts the estimated nonparametric functions of near heterophoria with penalized thin-plate regression splines. The relationship between the degree of near heterophoria and SE or AL could be described as an approximately parabolic curve. When the value of near heterophoria was less than 0 PD, SE increased as near heterophoria increased, while AL decreased as near heterophoria increased. In contrast, when the value of near heterophoria was greater than 0 PD, SE decreased as near heterophoria increased, while AL increased as near heterophoria increased. Thus, the two estimated curves indicated that greater degrees of near heterophoria (exophoria or esophoria) were associated with longer AL and more severe myopia. Model 1 was built using SE or AL as the dependent variable and near heterophoria as the independent variable.

In this study, we observed a weak correlation between near heterophoria and age (correlation coefficient, *r* = −0.082, *P* < 0.001). Although age adjustment in model 2 decreased the correlation between near heterophoria and AL, the correlation between the two variables remained significant (*P* < 0.01) (Table 4).

## 4. Discussion

This school-based survey was initiated in 2017 to investigate the associations of near heterophoria with AL and refraction among school children in Tianjin, China. Interestingly, our findings indicated that exophoria was the predominant type of near heterophoria in school-aged Chinese children. The prevalence of exophoria also increased as grade level increased. Notably, the present study was the first to report that greater degrees of near exophoria or esophoria were associated with longer AL and more severe myopia.

In this study, the most common type of near heterophoria in Chinese school children was exophoria (64.69%), irrespective of ametropia or age, occurring slightly more frequently than in previous studies [20, 22, 23]. Hong et al. observed exophoria in 21.50% of their study population, whereas Leone et al. noted that it accounted for 58.3% of cases in 6-year-old children and 52.2% of cases in 12-year-old children [20, 22]. In a study of children aged 8–13 years, Jang et al. reported that 39.0% (53/136), 38.2% (52/136), and 22.8% (31/136) of children exhibited exophoria between 0 and 6 PD, esophoria, and exophoria >6 PD, respectively, indicating that exophoria >2 PD (the standard used in the present study) accounted for more than 39.0% of cases [23]. The average degrees of exophoria in our study were 3.38 PD and 4.76 PD for children in grades 1-2 (around 6 years old) and grades 5-6 (around 12 years old), which were close to the values reported by Leone et al. (3.90 PD and 4.90 PD for 6- and 12-year-old children) [22].

Direct comparisons of our findings to those of previous studies were difficult given differences in region, myopia definitions, and measurement methods [28, 29]. In some studies, near heterophoria was measured at full prescription using the von Graefe technique, which likely stimulated short-term lens-induced changes in near heterophoria, resulting in an esophoric shift [23, 30]. The state of correction of the refractive error would affect the degree of heterophoria. Since a large proportion of children in China had undercorrected or uncorrected refractive error [31, 32], it was necessary to explore the heterophoria under this circumstance. In the present study, we adopted the method used by Leone et al. to assess near heterophoria, which was measured with children wearing their typical glasses or without the lens. This might explain why our results were more similar to those reported by Leone et al. [22].

The children were divided into three groups according to grades (grades 1-2, grades 3-4, and grades 5-6). Different grades had different learning environments and learning tasks, which had different requirements for vision. These differences may cause variable near heterophoria and refraction. However, because the age of primary school entrance varied greatly, the ages of the two adjacent grades might overlap, so the children from the two adjacent grades were put together to analyze.

Notably, both the proportion and value of exophoria increased as grade level increased in our study. Anderson et al. reported greater near exophoria among children with myopia assessed over a 10-year follow-up period, which might help to explain this finding [33]. Previous studies have also indicated that near heterophoria, especially esophoria, might be associated with the development and progression of myopia [14–18]. Consistent with these findings, our results indicated that greater degrees of esophoria were associated with longer AL and a higher degree of myopia. Moreover, we observed the same associations as the degree of exophoria increased.

Although analyses were adjusted for age to eliminate its confounding effects on AL and binocular vision, near heterophoria and noncycloplegic refraction remained significantly associated in the adjusted model. These results highlighted the need for further research to determine the exact mechanisms by which myopia develops and progresses in children with near heterophoria.

The present study had some limitations, including regional bias, as all participants attended elementary schools in Tianjin. In addition, noncycloplegic autorefraction measurements were used for analysis. Although such measurements had exhibited good consistency with other values obtained in large-scale refraction screening of the population [34, 35], they were not currently considered an appropriate substitute for cycloplegic refraction measurements. It was reported that myopia could be classified by the AL/corneal radius ratio, with higher values associated with more myopic refraction [36, 37]. We would seriously consider this as a way to define myopia in our future studies since the corneal curvature was not available in this study. Finally, since this was a cross-sectional study, we were unable to assess changes over time, highlighting the need for prospective longitudinal studies to determine the association of near heterophoria with axial elongation and myopia progression.

## 5. Conclusions

This study demonstrated that near heterophoria exhibited a significant correlation with refraction and AL. The most frequent type of near heterophoria in Chinese elementary school children was exophoria. Greater degrees of near heterophoria were associated with longer AL and a higher degree of myopia, irrespective of age. These findings highlighted the need to investigate the influence of near heterophoria on visual development in large-scale studies with longer follow-up periods.

## Figures and Tables

**Table 1:** Basic demographic parameters between different subjects.

	Included subjects (*n* = 11013)	Excluded subjects (*n* = 4068)	*Z*/*χ*^2^	*P*
Age (years)	9.26 ± 1.73	8.90 ± 1.55	−11.493	＜0.001^a^
Gender (male, %)	5826 (52.60%)	2256 (55.46%)	7.804	0.005^b^
SE (D)	−1.15 ± 1.61	−0.93 ± 2.12	−7.838	＜0.001^a^
AL (mm)	23.50 ± 1.09	23.28 ± 1.11	−5.720	＜0.001^a^
Heterophoria (PD)	−3.99 ± 6.03	−3.99 ± 6.09	−1.291	0.197^a^

^a^A significant difference between included and excluded subjects as determined using the Kruskal–Wallis test. ^b^A significant difference between included and excluded subjects as determined using the chi-square test.

**Table 2:** Number and proportion of near heterophoria cases among children with different refraction.

	Total	Exophoria	Orthophoria	Esophoria
	*N*	*N* (%)	*N* (%)	*N* (%)
*Grades * ***1-2***				
Emmetropia	2524	1592 (63.07)	692 (27.42)	240 (9.51)
Myopia	1163	719 (61.82)	270 (23.22)	174 (14.96)
Hyperopia	36	17 (47.22)	13 (36.11)	6 (16.67)

*Grades * ***3-4***				
Emmetropia	1726	1138 (65.93)	383 (22.19)	205 (11.88)
Myopia	2085	1346 (64.56)	505 (24.22)	234 (11.22)
Hyperopia	35	17 (48.57)	7 (20.00)	11 (31.43)

*Grades * ***5-6***				
Emmetropia	735	465 (63.27)	179 (24.35)	91 (12.38)
Myopia	2685	1851 (68.94)	509 (18.96)	325 (12.10)
Hyperopia	24	9 (37.50)	8 (33.33)	7 (29.17)

**Table 3:** SE and AL in children with different types of near heterophoria.

Heterophoria	N	SE	AL
Exophoria	7154	−1.20 ± 1.63^a^	23.53 ± 1.11^c^
Orthophoia	2566	−0.97 ± 1.45	23.42 ± 1.05
Esophoria	1293	−1.22 ± 1.74^b^	23.53 ± 1.10^d^
*H*/*F*		21.328	9.098
*P* value		<0.001	<0.001

^a^A significant difference between exophoria and orthophoria as determined using the Kruskal–Wallis test. ^b^A significant difference between orthophoria and esophoria as determined using the Kruskal–Wallis test. ^c^A significant difference between orthophoria and esophoria as determined by an analysis of variance (ANOVA). ^d^A significant difference between orthophoria and esophoria as determined by an analysis of variance (ANOVA).

**Table 4:** Generalized additive model analysis of the effect of near heterophoria on SE or AL.

	Linear		Nonlinear	
	*β*	*P* value	*β*	*P* value
*Near heterophoria, SE*				
Model 1	0.021	<0.001	1.000	<0.001
Model 2	0.012	<0.001	1.000	<0.001

*Near heterophoria, AL*				
Model 1	−0.016	<0.001	1.000	<0.001
Model 2	−0.008	<0.001	1.000	0.004

## Data Availability

The datasets generated during and/or analyzed during the current study are available from the corresponding author on reasonable request.
